# Characterization of age-related gene expression profiling in bone marrow and epididymal adipocytes

**DOI:** 10.1186/1471-2164-12-212

**Published:** 2011-05-05

**Authors:** Li-Fen Liu, Wen-Jun Shen, Masami Ueno, Shailja Patel, Fredric B Kraemer

**Affiliations:** 1 Division of Endocrinology, Stanford University, Stanford, CA, 94305-5103, USA; 2 VA Palo Alto Health Care System, Palo Alto, California, 94304, USA

## Abstract

**Background:**

While an increase in bone marrow adiposity is associated with age-related bone disease, the function of bone marrow adipocytes has not been studied. The aim of this study was to characterize and compare the age-related gene expression profiles in bone marrow adipocytes and epididymal adipocytes.

**Results:**

A total of 3918 (13.7%) genes were differentially expressed in bone marrow adipocytes compared to epididymal adipocytes. Bone marrow adipocytes revealed a distinct gene profile with low expression of adipocyte-specific genes peroxisome proliferator-activated receptor gamma (PPARγ), fatty acid binding protein 4 (FABP4), perilipin (Plin1), adipsin (CFD) and high expression of genes associated with early adipocyte differentiation (CCAAT/enhancer binding protein beta (C/EBPβ), regulator of G-protein signaling 2 (RGS2). In addition, a number of genes including secreted frizzled related protein 4 (SFRP4), tumor necrosis factor α (TNFα), transforming growth factor beta 1(TGFβ1), G-protein coupled receptor 109A (GPR109A) and interleukin 6 (IL-6), that could affect adipose-derived signaling to bone are markedly increased in bone marrow adipocytes. Age had a substantial effect on genes associated with mitochondria function and inflammation in bone marrow adipocytes. Twenty seven genes were significantly changed with age in both adipocyte depots. Among these genes, IL6 and GPR109A were significantly reduced with age in both adipocyte depots.

**Conclusions:**

Overall, gene profiling reveals a unique phenotype for primary bone marrow adipocytes characterized by low adipose-specific gene expression and high expression of inflammatory response genes. Bone marrow and epididymal adipocytes share a common pathway in response to aging in mice, but age has a greater impact on global gene expression in epididymal than in bone marrow adipocytes. Genes that are differentially expressed at greater levels in the bone marrow are highly regulated with age.

## Background

Aging is associated with impaired adipogenesis in various fat depots in humans [1-4]. With age and age-related osteoporosis, there is an inverse relationship between bone mass and bone marrow adiposity [5-7]. There are generally considered to be two types of adipose tissue, white and brown, both of which are able to store lipid but have different roles in energy metabolism [8,9]. Moreover, there are regional differences in the function among various adipose tissue depots; in humans visceral obesity presents a greater risk for obesity-related metabolic disease than subcutaneous obesity [10,11].

Previous functional studies of marrow adipocytes have mostly been limited to developmental studies [12]. Some studies have suggested that the presence of adipocytes can influence differentiation of mesenchymal stem cells (MSCs) into adipocytes, thereby inhibiting the differentiation into other cell lines [13]. We and others reported that directly co-culturing bone marrow MSCs with fully differentially adipocytes decreased osteoblast differentiation by decreasing RunX2 mRNA expression [14,15], suggesting that these cells are metabolically active but negatively regulate differentiation of MSCs into osteoblasts. Recent studies have suggested that, in addition to adipose, liver and muscle tissue, the osteoblast is also an important target tissue for insulin action [16,17]. Infiltration of fat in bone marrow could affect osteoblast function and differentiation through paracrine/endocrine effects of secretory products and adipocytokines [18,19]. Thus, bone marrow adipocytes might play a pivotal role in mediating the regulation of osteoblast function in aging and in diabetic or obese animals. A recent report demonstrated that bone marrow-derived adipocytes are distinct from epididymal white adipocytes [20]. While it is well known that ectopic fat accumulation in non-adipose tissues is greatly associated with age-related insulin resistance and metabolic disorders [21], the relationship of bone marrow adiposity with age-related diseases is unclear. In view of the specialized environment within the bone marrow with both active hematopoiesis and osteoblastogenesis ongoing, we hypothesized that adipocytes within the bone marrow might constitute a unique depot. In order to obtain a comprehensive understanding of the characteristics of bone marrow adipocytes, we profiled the gene expression patterns in bone marrow adipocytes with age and simultaneously examined differential gene expression in bone marrow and epididymal adipocytes with age. This study is the first to characterize primary bone marrow adipocytes and to demonstrate the effects of aging on two different adipocyte populations within the same animal. Our results demonstrate that while bone marrow adipocytes are distinct from epididymal white adipocytes, they also share a common inflammatory pathway in response to aging.

## Results

**Figure 1:**
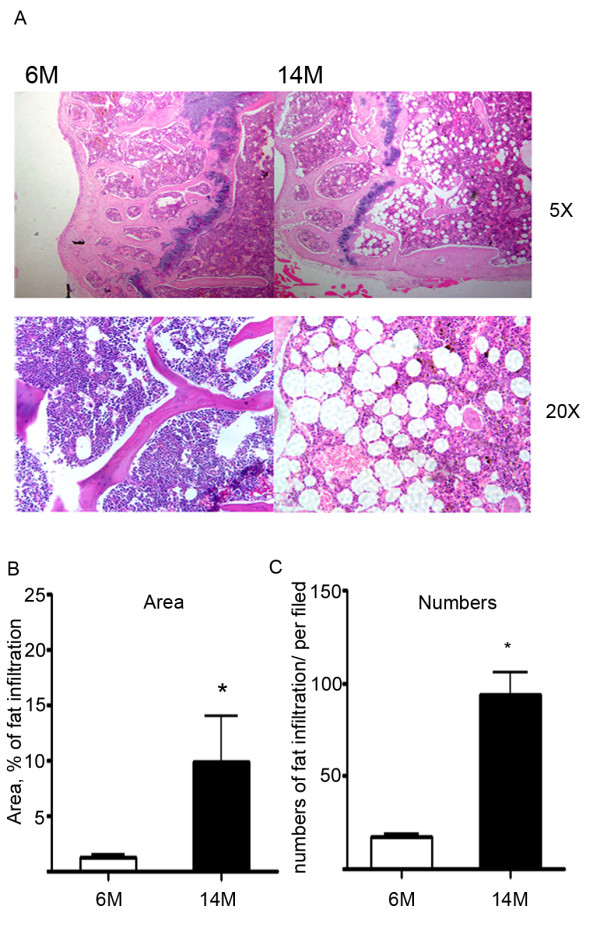
**Quantitative analysis of bone marrow adipocytes**. A. The top panels are images of the distal femurs from 6-month-old and 14-month-old C57BL6/J male mice stained with H&E (magnification ×5). The bottom panels are sections stained with H&E showing the numerous large adipocytes in bone marrow from distal femurs (magnification ×20). B. Area of bone marrow fat infiltration as a % of total area. C. Numbers of bone marrow fat cells (numbers/mm^2 ^bone marrow). Fields were taken from distal femur sections of 6-month-old and 14-month-old mice and calculated using ImagePro software.

Table 1 depicts several metabolic and biochemical parameters of the mice included in these studies. The metabolic parameters showed the expected negative effects of age on mice. Thus, there was a significant increase in weight and serum insulin and glucose levels in older mice compared to 6 month old animals. These negative metabolic effects of age were accompanied with significant increases in fat infiltration into bone marrow, as shown in Figure 1. Similarly, there were significant increases in leptin and adiponectin with age along with a significant decrease in circulating osteocalcin with age, whereas resistin, RANKL (receptor activator for nuclear factor κB ligand) and osteoprotegerin showed no changes with age.

**Table 1:** Metabolic parameters in aging mice

Parameter		6-month	14-month
	Weight (g)	35.0 ± 1.2	41.8 ± 4.0*
	Insulin (ng/mL)	0.57 ± 0.16	2.09 ± 0.44*
	Glucose (mg/dL)	118.7 ± 12	192.7 ± 17*
	Triglyceride (mg/dL)	240.9 ± 3.43	250.7 ± 6.8
Adipokines			
	Leptin (ng/mL)	3.52 ± 0.77	20.7 ± 2.38*
	Resistin (ng/mL)	1.35 ± 0.17	1.85 ± 0.20
	Adiponectin (μg/mL)	2.90 ± 0.20	5.66 ± 0.74*
Bone panel			
	Osteocalcin (ng/mL)	54.4 ± 4.8	31.8 ± 6.8*
	RANKL (ng/mL)	0.21 ± 0.03	0.17 ± 0.22
	Osteoprotegerin (ng/mL)	2.28 ± 0.40	1.92 ± 0.17

Data are mean ± SE of 6 to 10 mice per age group. Body weights were determined at time of necropsy. Serum metabolites were measured in the fed state. * *P *< 0.05 vs. 6-month-old.

### Adipocyte-specific genes in bone marrow adipocytes

**Figure 2:**
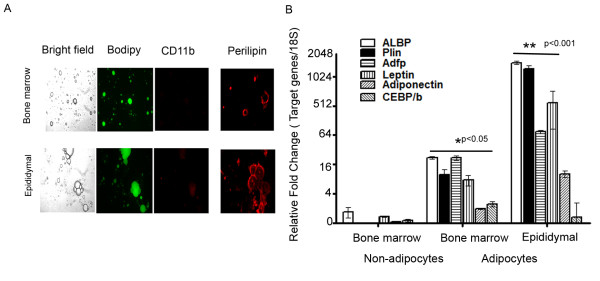
**Characterization of adipocyte preparations isolated from bone marrow and epididymal depots**. A. Light and immunofluorescence microscopy of adipocytes isolated from bone marrow and epididymal white adipose tissue. Fixed cells were incubated with either BODIPY 493/503 (1:500) or PE (phycoerythrin) conjugated anti-mouse CD11b (Intergrin a, Mac-1α) and polyclonal anti rabbit perilipin antibodies (1:200) in 1% blocking solution for 1 h. Following 1 h incubation of Alexa 555 (red) conjugated secondary antibodies at a dilution of 1/800 at room temperature, the stained cells were washed three to four times with PBS and observed using a Zeiss microscope observer A1. B. Expression of selected adipose genes in preparations of bone marrow cells, adipocytes isolated from bone marrow and adipocytes isolated from epididymal adipose tissue. A set of original RNA from the same animal was re-amplified to aRNA, then converted to cDNA. Relative fold change was normalized to endogenous 18S and bone marrow stromal cells. Data are presented as Log2 of fold change.

Bone marrow adipocytes were isolated by flushing out bone marrow cells from femurs and tibias and then isolating the adipocytes by flotation. White adipocytes were isolated from epididymal adipose tissue by flotation following collagenase digestion. Figure 2A displays the characteristics of the adipocyte cell preparations from bone marrow and epididymal depots. Epididymal adipocytes tended to be larger, but both adipocyte populations stained neutral lipids with Bodipy. In order to evaluate the degree of contamination of the cell preparations with monocyte/macrophages, the percentage of monocytes present in the samples was determined by identifying the number of CD11b positive cells. Approximately 10-17% of cells were identified as CD11b positive in both bone marrow and epididymal white adipocyte preparations (Figure 2A). In addition, the adipocytes from both bone marrow and epididymal depots stained with perilipin, but epididymal adipocytes had greater immunostaining. We verified the expression of a select group of adipocyte specific genes in the isolated adipocytes by RT-PCR (Reverse transcription polymerase chain reaction). FABP4 (fatty acid binding protein 4, adipocyte), perilipin (Plin1), ADFP (Plin2), leptin, adiponectin and C/EBPβ (CCAAT/enhancer binding protein beta) were all much more highly expressed in bone marrow adipocytes compared to the non-floating bone marrow stromal cells, as would be expected for adipocyte specific genes (Figure 2B). However, all of these genes, except C/EBPβ, were expressed at relatively lower levels in bone marrow compared to epididymal adipocytes. The differences in expression levels between bone marrow and epididymal adipocytes determined by qRT-PCR were mostly in agreement with the microarray data obtained (see below).

### Global characteristics of bone marrow adipocytes

**Figure 3:**
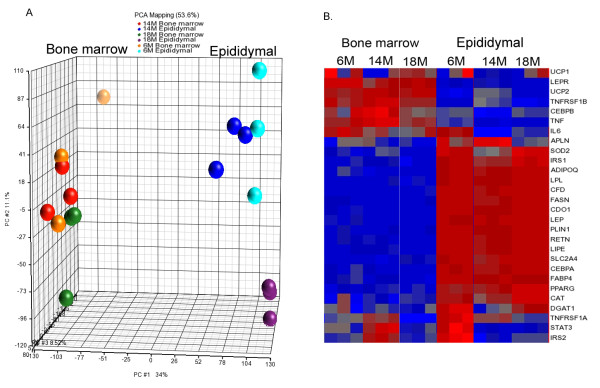
**Bone marrow adipocytes are distinct from epididymal adipocytes**. Bone marrow adipocytes and epididymal adipocytes were isolated from the same mice (age 6-month, 14-month and 18-month-old). Cells from several animals were pooled to yield sufficient sample to analyze three arrays for each age group and both adipocyte populations. A total of 18 arrays were included in the microarray analysis. A. Principal component analysis of array data before normalization showing clustering of bone marrow adipocytes as separate from epididymal adipocytes. B. Supervised hierarchical cluster analysis of 27 genes in adipocyte differentiation following normalization of all data sets.

We analyzed the differential expression of adipocyte genes isolated from bone marrow and epididymal fat depots from adult (6-month-old) and aged mice (14-and 18-month-old) using microarrays. Inspection of the frequency of expression values after normalization of the data showed consistent values between all samples (Additional File 1, Figure S1). Principal component analysis revealed a visible separation between bone marrow and epididymal adipocytes (Figure 3A). Moreover, there is a clear separation between epididymal adipocytes from 18-month and younger mice. This clustering represents the overall expression patterns, but does not provide the expression of individual genes. Analysis of genes based on functional category was performed to measure the differences and the similarities between the two adipocyte populations. A total of 3918 (13%) of the 28853 well-characterized mouse genes were differentially expressed between the two adipocyte populations. This selection was based on adjusted p < 0.05 and a fold change ± 2.0. These 3918 genes were assigned to biologically meaningful gene ontology (GO) categories. Differences in gene expression between bone marrow and epididymal adipocytes were primarily observed in three categories: biological process, molecular function and cellular component (Table 2). Subcategories of biological process include regulation of cell cycle, cell death and cell differentiation and regulation of metabolic processes, for instance carbohydrate and lipid metabolism, whereas molecular function includes genes associated with protein binding, enzyme activity and transcription regulator activity, and cellular component includes genes that are associated with the cell membrane, extracellular matrix, synapses and membrane bound organelles. The table displays the enrichment score and *p*-value in subcategories of the overall category of biological processes that are most relevant to adipocyte-specific functions, whereas the subcategories of cellular component and molecular function are not shown because the *p*-value of these overall categories did not reach statistical significance. As shown in Table 2, fat cell differentiation comprised 71-74% of genes in the ontology functional analysis. We next loaded this set of 3918 genes differentially expressed in the bone marrow and epididymal adipocytes into Ingenuity Pathway Analyses for further biological functional analysis. A set of 277 genes was generated by filtering expression in adipose tissue. This set of genes displayed a substantial differential expression pattern between bone marrow and epididymal adipocytes (Table 3). Table 3 displays the genes that were most differentially expressed and that are associated with network and biological pathways in adipocytes. Of these 277 genes, supervised hierarchical clustering of 28 genes that are associated with the adipocyte phenotype revealed that all were expressed in bone marrow adipocytes and that their expression patterns differed markedly from epididymal adipocytes (Figure 3B). In contrast to Figure 3A, there appears to be a separation between 6-month-old and elder mice in expression of some of these adipose-specific genes in epididymal adipocytes in Figure 3B; however, it is important to note that Figure 3A represents global, not individual, gene differences.

**Table 2:** Gene ontologies with differentially expressed genes in bone marrow adipocytes

Functional Group	Enrichment Score*	*P *value**	% genes in group present
Biological process	4.39	0.01	18.10
Cellular	40.9	<0.001	21.9
Immune system	18.36	<0.001	31.7
Metabolic	16.67	<0.001	20.7
Oxidation reduction	37.36	<0.001	30.52
Lipid	23.63	<0.001	34.7
Carbohydrate	7.41	<0.001	28.05
Developmental	4.3	0.01	19.71
Adipocyte differentiation	18.3	<0.001	47.27
Brown	31.44	<0.001	74.07
White	8.46	<0.001	71.43
Cellular component	3.55	0.03	17.97
Molecular component	1.80	0.17	17.91

A selection of genes generated from ANOVA analysis was assigned to biologically meaningful gene ontology (GO) categories using GO enrichment analysis (Partek^® ^Genome Suite software, version 6.3). Listed are groups of gene-associated biological processes in bone marrow adipocytes compared with epididymal adipocytes (fold change cut off is 2.0). *A high value of enrichment score indicates that the functional group is over-represented in the gene list. **P value is the -log *p*-value of a Chi-square test.

**Table 3:** Comparison of adipocyte-genes between bone marrow and epididymal adipocytes

Symbol	Entrez Gene Name	6-month	14-month	18-month
**Increased expression in bone marrow**				
LEPR	leptin receptor	19.7	7.4	16.4
OSM	oncostatin M	18.1	20.1	11.4
RGS2	regulator of G-protein signaling 2, 24kDa	15.3	15.8	8.9
SFRP4	secreted frizzled-related protein 4	14.8	1.8	6.6
ABCB4	ATP-binding cassette, sub-family B, member 4	10.7	6.9	7.4
TNFRSF1β	*CD120b*	6.6	4.3	8.3
TNFα	tumor necrosis factor α	5.0	26.3	7.2
100001G20RIK	RIKEN cDNA 1100001G20 gene, Wdnm1-like	4.2	4.5	3.0
UCP2	uncoupling protein 2	4.0	2.1	3.7
LPIN2	lipin 2	4.0	3.9	5.6
TGFB1	transforming growth factor, beta 1	3.9	2.5	3.8
GPR109A	G protein-coupled receptor 109A	3.7	33.6	2.3
C/EBPβ	CCAAT/enhancer binding protein beta	2.3	6.8	1.3
IL6	interleukin 6	-1.1*	8.8	2.8

**Decreased expression in bone marrow**				
LEP	leptin	-152.5	-300.1	-454.4
CFD	complement factor D (adipsin)	-138.2	-115.9	-107.1
RETN	resistin	-114.7	-87.4	-153.7
CDO1	cysteine dioxygenase, type I	-106.9	-132.3	-192.1
PLIN1	perilipin 1	-100.6	-85.1	-106.5
ERPINE1	plasminogen activator inhibitor type 1	-60.9	-7.0	-19.0
FASN	fatty acid synthase	-32.9	-28.5	-43.5
ADIPOQ	adiponectin	-26.4	-35.3	-41.2
FABP4	fatty acid binding protein 4, adipocyte	-23.2	-27.0	-41.3
LIPE	lipase, hormone-sensitive	-21.9	-15.2	-26.8
LPL	lipoprotein lipase	-21.8	-18.8	-23.9
KLB	klotho beta	-21.1	-12.9	-29.6
ACOX1	acyl-Coenzyme A oxidase 1, palmitoyl	-16.5	-10.2	-12.8
FGFR1	fibroblast growth factor receptor 1	-12.2	-11.4	-10.2
SLC2A4	glucose transporter member 4	-10.2	-9.5	-9.2
C/EBPα	CCAAT/enhancer binding protein, alpha	-6.7	-8.7	-11.2
PPARγ	peroxisome proliferator-activated receptor gamma	-5.9	-4.8	-10.3
IDH1	isocitrate dehydrogenase 1 (NADP+), soluble	-5.1	-4.7	-6.4
ADIPOR2	adiponectin receptor 2	-4.7	-4.0	-4.7

The list includes the most differentially expressed genes in bone marrow adipocytes compared to epididymal adipocytes. The set of the significantly differentially expressed genes was selected on the basis of their expression in adipose cells according to the IPA knowledge-base. For each gene, the fold change value in gene expression was calculated between mean values in bone marrow adipocytes versus epididymal adipocytes in mice (6-month, 14-month and 18-month old). The significance of differences was measured by one-way ANOVA. All values are *p *< 0.01 except * *P *> 0.01

### Functional analysis of differential gene expression between bone marrow and epididymal white adipocytes

**Figure 4:**
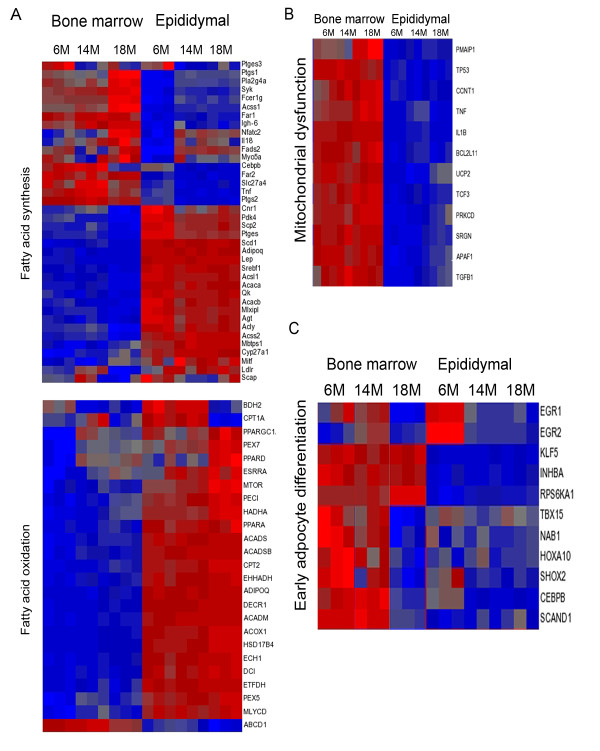
**Heat maps of genes differentially expressed in bone marrow adipocytes**. A. Clustering of genes involved in lipid metabolism (fatty acid synthesis, upper panel, and fatty acid oxidation, lower panel) with at least a 2-fold difference between bone marrow adipocytes compared with epididymal adipocytes. B. Clustering of genes involved in decreased transmembrane potential of mitochondria with at least a 2-fold difference between bone marrow adipocytes compared with epididymal adipocytes. C. Clustering of genes involved in early adipocyte differentiation with at least a 2-fold difference between bone marrow adipocytes compared with epididymal adipocytes.

We further analyzed the over-represented 3918 genes (2-fold change) by GO enrichment (Partek) and Ingenuity Pathway analyses. Among these genes, a set of 1940 genes was decreased greater than 2-fold (*p *≤ 0.05) in bone marrow adipocytes compared with epididymal adipocytes across age. Of these 1940 genes, there were 547 genes associated with bio-functional groups and networks. This set of genes was enriched and associated with lipid metabolism, molecular transport and small molecule biochemistry. Figure 4A shows a supervised hierarchal cluster of genes related to adipocyte lipid metabolism, including fatty acid synthesis (upper panel) and oxidation (lower panel), that were 2-fold lower in bone marrow adipocytes. This gene set included leptin, Acsl1 (acyl-coA synthetase 1), adiponectin and Scap (SREBF chaperone) in fatty acid synthesis and PPARα, PGC1α, CPT1α and ACOX1 in fatty acid oxidation. Clustering also highlighted a group of 1965 genes that were increased 2-fold or more in bone marrow adipocytes. Of these 1965 genes, there were 337 genes associated with biological function networks. These genes are associated with pro-apoptosis, pro-inflammatory cytokines and mitochondrial dysfunction. As listed in Table 3, genes displaying increased expression in bone marrow adipocytes include leptin receptor (Lepr), G-protein coupled receptor 109A (GPR109A) and gp-130 cytokines, oncostatin M (OM), interleukin 6 (IL-6) and TNFα. Adipocyte specific genes (PPARγ, FABP4, perilipin, adipsin and leptin) were expressed at significantly lower levels in bone marrow adipocytes compared with epididymal adipocytes. Figure 4B shows a supervised hierarchal cluster of 11 genes associated with decreases in transmembrane potential of mitochondria and apoptosis. These genes included p53, IL1β (interleukin 1β), BCL2L11 (BCL2-like 11, apoptosis facilitator protein), UCP2 and TGFβ1. Figure 4C shows a supervised cluster of 11 genes associated with early adipocyte differentiation. These genes include EGR1 (early growth response 1, Krox-1), EGR2 (early growth response 2, Krox-20), KLF5 (Kruppel-like factor 5), INHBA (inhibin beta A, activin), CEBP/β and RPS6KA1 (ribosomal protein S6 kinase 1).

### Age-related alteration of gene expression in bone marrow and epididymal white adipocytes

**Figure 5:**
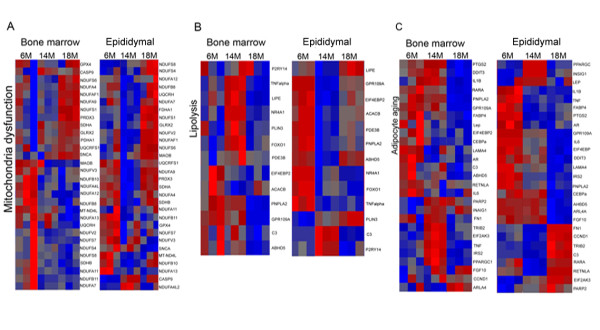
**Age-related gene expression in adipocytes**. Heat maps of age-associated gene expression changes in bone marrow and epididymal white adipocytes. A. Clustering of genes involved in mitochondria function. B. Clustering of genes associated with lipolysis. C. Clustering of age-regulated genes in bone marrow and epididymal adipocytes.

We next sought to identify genes affected by aging within each adipocyte population based on ontology. Aging-related alterations of gene expression were identified and a list of genes was generated yielding a total of 5649 genes that displayed significant changes in both bone marrow and epididymal adipocytes with age. Analyses were conducted using two-way ANOVA including interaction of cell types and age. The comparisons were conducted by comparing 14-month-old and 18-month-old to 6-month-old. Genes were further analyzed by Ingenuity Pathway Analyses to relate age-associated gene expression changes to biological function and signaling pathways. Age-related gene changes in adipocytes fell into several categories of biological function including inflammatory response, genetic disorder and cellular development, whereas the majority of alterations of genes in both adipocyte depots were associated with mitochondrial dysfunction and lipid metabolism. Figure 4A shows that genes involved in fatty acid synthesis were increased with age in bone marrow adipocytes, including Acss1 (acyl-coA synthetase short chain family 1), Fads2 (fatty acid desaturase 2), and Slc27a4 (fatty acid transporter 4). Heat maps of age-altered genes in the bone marrow and epididymal adipocytes are indicated in Figure 5. Figure 5A illustrates age-related changes in genes involved in mitochondria function in which 18-month-old bone marrow adipocytes and epididymal adipocytes were most affected. Figure 5B displays the differential expression of genes involved in lipolysis in response to aging in both adipocyte populations. TNFα, Plin3 and Lipe (hormone sensitive lipase) increased in 14-month-old bone marrow adipocytes, whereas most of these genes decreased in 14-month-old epididymal adipocytes. PNPLA2 (ATGL) was decreased with age in both adipocyte depots. As shown in Table 4, pathways and networks associated with adipocyte differentiation, lipolysis and mitochondria function displayed significant changes with age in adipocytes. Approximately 60 percent of genes associated with adipocyte differentiation were significantly up-regulated in 14-month-old bone marrow adipocytes, whereas 70 percent of genes were down-regulated in 14-month-old epididymal adipocytes. A similar pattern was also observed in lipolysis pathways. In addition, a group of genes associated with mitochondria function was significantly regulated in bone marrow adipocytes with age, but not in epididymal adipocytes, as indicated in Table 4. Fold change values of genes involved in adipocyte differentiation, lipolysis and mitochondrial function are listed in Tables 5, 6 and 7.

**Table 4:** Top regulated pathways in bone marrow and epididymal adipocytes with age

			Genes		Genes assigned		
				
Pathway	Cell type	Age (M)	Up	Down	In pathway	Ratio*	-Log10 (*p *value)**
Adipocyte differentiation	Bone marrow	14M	18	12	30	0.36	1.922
		18M	11	19		0.486	4.436
	Epididymal	14M	9	21	30	0.61	6.019
		18M	8	22		0.46	2.498

Lipolysis	Bone marrow	14M	6	7	13	0.289	1.347
		18M	5	8		0.263	0.87
	Epididymal	14M	2	11	13	0.342	1.203
		18M	2	11		0.289	0.659

Mitochondria dysfunction	Bone marrow	14M	12	18	30	0.237	1.115
		18M	13	17		0.323	3.364
	Epididymal	14M	3	27	30	0.24	0.487
		18M	21	9		0.226	0.28

For each gene, the fold change of expression of 14-month-old (14M) and 18-month-old (18M) was calculated versus 6-month-old (6M). Pathways that have significant association with the whole set of regulated genes in bone marrow and epididymal adipocytes with age were assessed using IPA software. * Ratio of altered genes in bone marrow adipocytes to assigned pathway. **The p-value was calculated by IPA using Fisher's Exact Test. A threshold of 1.30 was selected (p < 0.05)

**Table 5:** Age-associated fold changes in genes involved in adipocyte differentiation

		Bone marrow		Epididymal	
Symbol	Entrez Gene Name	14M	18M	14M	18M
AR	androgen receptor	-1.84	-2.03	-1.47	-1.82
ARL4A	ADP-ribosylation factor-like 4A	-1.34	1.28	-2.04	1.03
CCND1	cyclin D1	1.26	1.11	2.48	-1.64
CEBPA	CCAAT/enhancer binding protein (C/EBP), alpha	-1.51	-1.43	-1.33	1.15
DDIT3	DNA-damage-inducible transcript 3	1.22	-2.14	-1.76	-1.16
EBF1	early B-cell factor 1	1.43	-1.50	-1.28	-1.41
EIF2AK3	eukaryotic translation initiation factor 2-alpha kinase 3	1.80	1.17	1.70	1.53
FABP4	fatty acid binding protein 4, adipocyte	-1.54	-2.57	-1.23	-1.32
FGF10	fibroblast growth factor 10	1.23	1.18	-2.60	1.04
FN1	fibronectin 1	1.36	-1.18	7.06	-3.16
IL6	interleukin 6 (interferon, beta 2)	-2.04	-2.07	-20.53	-5.56
IL1B	interleukin 1, beta	1.37	-1.48	-2.26	-2.16
INSIG1	insulin induced gene 1	1.26	1.49	1.33	2.81
IRS2	insulin receptor substrate 2	1.96	-1.13	-4.26	-2.94
LAMA4	laminin, alpha 4	-1.44	-1.38	-1.41	-1.18
LEP	leptin	-2.02	-1.77	1.01	1.59
LIF	leukemia inhibitory factor (cholinergic differentiation factor)	1.88	1.11	-1.03	-1.07
NR3C1	nuclear receptor subfamily 3, group C, member 1 (glucocorticoid receptor)	1.29	1.02	1.01	1.15
PARP2	poly (ADP-ribose) polymerase 2	1.35	1.24	1.30	1.21
PPARGC1A	peroxisome proliferator-activated receptor gamma, coactivator 1 alpha	1.41	-1.09	-1.05	2.02
PTGS1	prostaglandin-endoperoxide synthase 1 (prostaglandin G/H synthase and cyclooxygenase)	-1.34	1.03	3.29	-1.59
PTGS2	prostaglandin-endoperoxide synthase 2 (prostaglandin G/H synthase and cyclooxygenase)	1.43	-2.09	-3.75	-5.58
RARA	retinoic acid receptor, alpha	-1.30	-2.35	1.06	-1.49
RETNLA	resistin like alpha	-1.32	-1.11	2.96	-10.77
SCD	stearoyl-CoA desaturase (delta-9-desaturase)	1.80	1.11	1.01	1.05
TNF	tumor necrosis factor	2.36	-1.48	-2.37	-2.18
TRIB2	tribbles homolog 2	2.98	1.17	1.78	-1.18
VDR	vitamin D (1,25- dihydroxyvitamin D3) receptor	-1.39	-1.32	-1.12	-1.21

The list includes age-related fold change in genes involved in adipocytes differentiation in bone marrow and epididymal adipocytes. The set of regulated genes was selected according to the IPA knowledge-base. The 14-month (14M) and 18-month-old (18M) were compared to 6-month-old. The significance of differences was measured by two-way ANOVA. The significance of differences was estimated by a Benjamini-Hochberg corrected p value (p < 0.01).

**Table 6:** Age-related fold changes in genes involved in lipolysis

		Bone marrow		Epididymal	
Symbol	Entrez Gene Name	14M	18M	14M	18M
ABHD5	αβ hydrolase domain containing 5	-1.12	-1.26	-1.97	1.04
ACACB	acetyl-CoA carboxylase beta	-1.40	-1.14	-2.22	-1.31
ADRB2	adrenergic, beta-2-, receptor, surface	1.22	1.49	-1.37	-2.04
C3	complement component 3	-1.07	-1.23	2.16	-1.11
EIF4EBP2	eukaryotic translation initiation factor 4E binding protein 2	-1.38	-1.53	-1.60	-1.15
FOXO1	forkhead box O1	1.99	1.17	-1.42	-1.44
GPR109A	G protein-coupled receptor 109A	-1.29	-3.99	-10.52	-2.20
NR4A1	nuclear receptor subfamily 4, group A, member 1	2.53	1.11	-4.03	-4.62
P2RY14	purinergic receptor P2Y, G-protein coupled, 14	1.07	1.05	1.54	-1.21
PDE3B	phosphodiesterase 3B, cGMP-inhibited	1.57	1.25	-1.99	-1.32
PLIN1	perilipin 1	-1.24	-1.21	-1.36	1.04
PNPLA2	patatin-like phospholipase domain containing 2	-1.03	-1.43	-1.39	-1.21
TNF	tumor necrosis factor	2.37	-1.38	-2.36	-2.12

The list includes age-related fold change in genes involved in lipolysis in bone marrow and epididymal adipocytes. The set of regulated genes was selected according to the IPA knowledge-base. The 14-month (14M) and 18-month-old (18M) were compared to 6-month-old. The significance of differences was measured by two-way ANOVA. The significance of differences was estimated by a Benjamini-Hochberg corrected p value (p < 0.01).

**Table 7:** Age-related fold changes in genes involved in mitochondrial function

		Bone marrow		Epididymal	
Symbol	Entrez Gene Name	14M	18M	14M	18M
CASP9	caspase 9, apoptosis-related cysteine peptidase	1.51	1.38	1.27	1.23
GLRX2	glutaredoxin 2	1.36	1.79	-1.05	1.38
GPX4	glutathione peroxidase 4 (phospholipid hydroperoxidase)	1.08	1.30	-1.12	-1.11
MAOB	monoamine oxidase B	-1.23	-1.39	-1.06	1.31
MT-ND4L	NADH dehydrogenase, subunit 4L (complex I)	-1.25	-1.63	-1.22	-1.17
NDUFA4	NADH dehydrogenase (ubiquinone) 1 alpha subcomplex, 4, 9 kDa	1.11	1.81	-1.21	1.00
NDUFA7	NADH dehydrogenase (ubiquinone) 1 alpha subcomplex, 7, 14.5 kDa	-1.72	-1.69	-1.15	1.12
NDUFA11	NADH dehydrogenase (ubiquinone) 1 alpha subcomplex, 11, 14.7 kDa	-1.46	-1.68	-1.07	1.04
NDUFA12	NADH dehydrogenase (ubiquinone) 1 alpha subcomplex, 12	-1.58	-1.71	-1.11	1.09
NDUFA13	NADH dehydrogenase (ubiquinone) 1 alpha subcomplex, 13	-1.05	-1.33	-1.26	-1.30
NDUFA4L2	NADH dehydrogenase (ubiquinone) 1 alpha subcomplex, 4-like 2	-1.26	-1.38	1.50	1.05
NDUFA9 (includes EG:4704)	NADH dehydrogenase (ubiquinone) 1 alpha subcomplex, 9, 39 kDa	1.11	1.77	-1.46	1.10
NDUFAF1	NADH dehydrogenase (ubiquinone) 1 alpha subcomplex, assembly factor 1	1.18	1.87	-1.41	1.42
NDUFB8	NADH dehydrogenase (ubiquinone) 1 beta subcomplex, 8, 19 kDa	-1.31	-1.40	-1.35	1.09
NDUFB10	NADH dehydrogenase (ubiquinone) 1 beta subcomplex, 10, 22 kDa	-1.46	-1.38	-1.18	1.01
NDUFB11	NADH dehydrogenase (ubiquinone) 1 beta subcomplex, 11, 17.3 kDa	-1.58	-1.38	-1.18	-1.13
NDUFS1	NADH dehydrogenase (ubiquinone) Fe-S protein 1, 75 kDa (NADH-coenzyme Q reductase)	1.29	2.22	-1.21	1.67
NDUFS4	NADH dehydrogenase (ubiquinone) Fe-S protein 4, 18 kDa (NADH-coenzyme Q reductase)	-1.22	-1.39	-1.03	1.48
NDUFS7	NADH dehydrogenase (ubiquinone) Fe-S protein 7, 20 kDa (NADH-coenzyme Q reductase)	-1.53	-1.51	-1.16	-1.08
NDUFS8	NADH dehydrogenase (ubiquinone) Fe-S protein 8, 23 kDa (NADH-coenzyme Q reductase)	-1.37	-1.52	1.14	1.46
NDUFS6 (includes EG:4726)	NADH dehydrogenase (ubiquinone) Fe-S protein 6, 13 kDa (NADH-coenzyme Q reductase)	1.01	1.68	-1.08	1.29
NDUFV2	NADH dehydrogenase (ubiquinone) flavoprotein 2, 24 kDa	-1.54	-1.31	-1.27	1.26
NDUFV3	NADH dehydrogenase (ubiquinone) flavoprotein 3, 10 kDa	-1.24	-1.38	-1.11	-1.12
PDHA1 (includes EG:5160)	pyruvate dehydrogenase (lipoamide) alpha 1	1.62	1.85	-1.20	1.45
PRDX3	peroxiredoxin 3	1.19	2.39	-1.42	1.19
SDHA	succinate dehydrogenase complex, subunit A, flavoprotein (Fp)	1.31	1.51	-1.35	1.06
SDHB	succinate dehydrogenase complex, subunit B, iron sulfur (Ip)	-1.25	-1.31	-1.35	1.10
SNCA	synuclein, alpha (non A4 component of amyloid precursor)	-1.08	1.67	-1.16	-3.22
UQCRFS1	ubiquinol-cytochrome c reductase, Rieske iron-sulfur polypeptide 1	1.20	1.49	-1.68	-1.05
UQCRH	ubiquinol-cytochrome c reductase hinge protein	-1.14	-1.32	-1.27	1.24

The list includes the mitochondrial genes that change with age in bone marrow and epididymal adipocytes. The set of regulated genes was selected on the basis of their expression in adipose cells according to the IPA knowledge-base. For each gene, the fold change value in gene expression was calculated between mean values in bone marrow adipocytes. The 14-month (14M) and 18-month-old (18M) were compared to 6-month-old. The significance of differences was measured by two-way ANOVA.

As indicated in Table 4, differential gene expression profiling was observed between bone marrow and epididymal adipocytes in pathway analysis. In order to examine the differences with age between bone marrow and epididymal adipocytes, we analyzed the fold changes with age in each adipocyte population separately. Table 8 shows the top-regulated adipocyte genes in bone marrow adipocytes with age. This list indicates that adiponectin and IL6 were the most down-regulated genes, while TNFα and genes associated with fatty acid transport and lipolysis, such as CD36 and Lipe (hormone sensitive lipase, HSL), were up-regulated. The increases in CD36 and HSL were not observed in epididymal adipocytes. On the other hand, Table 9 indicates that lipid droplet associated proteins Plin2 and Plin3 were significantly increased with age in epididymal white adipocytes, whereas Plin 3, but not Plin2, was significantly altered in bone marrow adipocytes. In addition, some adipocyte-specific genes (PPARγ, Plin3 and CEBPβ) increased in 14-month-old, then decreased in the 18-month-old group in bone marrow adipocytes, whereas they were not altered in the same direction in epididymal adipocytes (Tables 8 and 9).

**Table 8:** Top regulated adipocyte genes in bone marrow adipocytes with age

Symbol	Entrez Gene Name	14M	18M	*p*-value (cell type*age)
TNFα	tumor necrosis factor α	2.37	-1.38	1.47E-05
FOXO1	forkhead box O1	1.99	1.17	0.0006
IRS2	insulin receptor substrate 2	1.93	-1.08	0.0008
C/EBPβ	CCAAT/enhancer binding protein beta	1.79	-1.23	1.22E-05
CD36	fatty acid translocase	1.38	2.00	0.001
PPARγ	peroxisome proliferator-activated receptor gamma	1.36	1.08	0.003
PLIN3	perilipin 3, TIP47	1.25	1.04	0.007
SOD2	superoxide dismutase 2, mitochondrial	1.24	-1.09	0.001
UCP2	uncoupling protein 2	1.24	1.18	8.1E-06
LIPE	lipase, hormone sensitive	1.23	-1.27	2.1E-05
SERPINE1	plasminogen activator inhibitor type 1	1.21	-1.30	0.0004
GPX1	glutathione peroxidase 1	1.04	1.23	0.0002
SLC2A4	GLUT-4	-1.11	1.30	0.04
CFD	adipsin	-1.12	1.25	0.002
FABP4	fatty acid binding protein 4, adipocyte	-1.52	-2.46	0.001
PTPN11	protein tyrosine11	-1.58	-1.21	0.0006
ADIPOR2	adiponectin receptor 2	-1.66	-1.44	0.001
LEPR	leptin receptor	-1.94	1.44	0.002
IL6	interleukin 6 (interferon, beta 2)	-2.19	-1.91	8.34E-05
ADIPOQ	adiponectin	-2.25	-1.88	0.01

The list includes the adipocyte genes that change with age in bone marrow adipocytes. The set of regulated genes was selected on the basis of their expression in adipose according to the IPA knowledge-base. For each gene, the fold change value in gene expression was calculated between mean values in bone marrow adipocytes from mice. The 14-month (14M) and 18-month-old (18M) were compared to 6-month-old. The significance of differences was measured by two-way ANOVA.

**Table 9:** Top regulated adipocyte genes in epididymal adipocytes with age

	Entrez Gene Name	14M	18M	*P *value (cell type*age)
UCP2	uncoupling protein 2	2.3	1.3	8.1E-061
LEPR	leptin receptor	1.3	-1.1	0.02
FGFR1	fibroblast growth factor receptor 1	1.3	-1.5	0.03
PLIN3	perilipin 3, TIP47	1.2	1.6	0.0007
PPARγ	peroxisome proliferator-activated receptor gamma	1.1	1.8	0.0003
PLIN2	Perilipin 2, adipophilin, ADFP	1	1.7	5.8E-05
ADIPOQ	Adiponectin	-1.2	1.2	0.01
FABP4	fatty acid binding protein 4, adipocyte	-1.2	-1.3	0.05
C/EBPα	CCAAT/enhancer binding protein, alpha	-1.3	1.1	0.01
PPARGC1α	peroxisome proliferator-activated receptor gamma, coactivator 1 alpha	-1.5	2	1.4E-05
ADIPOR2	adiponectin receptor 2	-1.6	-1.1	0.001
SOD2	superoxide dismutase 2, mitochondrial	-1.8	-1.1	0.0001
TNFα	TNFα	-2.3	-2.1	0.0001
C/EBPβ	CCAAT/enhancer binding protein, beta	-2.4	-1	1.4E-05
IRS2	insulin receptor substrate 2	-4.3	-2.8	1.2E-05
SERPINE1	plasminogen activator inhibitor type 1	-7.3	-4.3	0.0004
IL6	interleukin 6	-19.8	-4.8	8.3E-05

The list includes the adipocyte genes that change with age in epididymal adipocytes. The set of regulated genes was selected on the basis of their expression in adipose according to the IPA knowledge-base. For each gene, the fold change value in gene expression was calculated between mean values in epididymal adipocytes. The 14-month (14M) and 18-month-old (18M) were compared to 6-month-old. The significance of differences was measured by two-way ANOVA.

Furthermore, in order to determine the most highly regulated adipocyte-specific genes in response to aging that are common to both adipocyte populations, we identified 27 genes in which expression levels were significantly altered with age in both adipocytes using a three-way ANOVA model with *p *< 0.05 in the interaction of age and cell types, as shown in Figure 5C and Table 10. A heat map of age-altered adipocyte genes (Figure 5C) reveals that 19 of 27 genes (70%) were regulated in the same direction with age in both adipocyte populations. These genes include EIF2AK3 (eukaryotic translation initiation factor 2-alpha kinase 3), FN1 (fibronectin 1), INSIG1 (insulin induced gene 1), PNPLA2, ABHD5, GPR109A, FABP4 and IL6 (Table 10). The other 8 genes were regulated with age in both adipocyte populations, but their patterns of regulation differed between bone marrow and epididymal adipocytes. Interestingly, the list indicates that IL6 (interleukin 6) was the most down-regulated gene with age in 14-month-old bone marrow and epididymal adipocytes, and GPR 109A (G protein-coupled receptor 109A) was significantly decreased 4.5 fold in 18-month-old bone marrow and reduced 10.6 fold in 14-month-old epididymal adipocytes compared to 6-month-old (Table 10).

**Table 10:** Age-regulated adipocyte gene expression

		Bone marrow		Epididymal		
			
Symbol	Entrez Gene Name	14M	18M	14M	18M	*p*-value (Age)
TRIB2	tribbles homolog 2 (Drosophila)	3.0	1.2	1.8	-1.2	1.78E-05
TNFα	tumor necrosis factor α	2.4	-1.5	-2.4	-2.2	0.00013
IRS2	insulin receptor substrate 2	2.0	-1.1	-4.3	-2.9	0.00018
EIF2AK3	eukaryotic translation initiation factor 2-alpha kinase 3	1.8	1.2	1.7	1.5	2.12E-06
PTGS2	prostaglandin-endoperoxide synthase 2 (prostaglandin G/H synthase and cyclooxygenase)	1.4	-2.1	-3.8	-5.6	4.87E-07
PPARGC1α	peroxisome proliferator-activated receptor gamma, coactivator 1 alpha	1.4	-1.1	-1.0	2.0	0.0024
IL1β	interleukin 1, beta	1.4	-1.5	-2.3	-2.2	0.00026
FN1	fibronectin 1	1.4	-1.2	7.1	-3.2	4.20E-07
PARP2	poly (ADP-ribose) polymerase 2	1.4	1.2	1.3	1.2	0.0018
CCND1	cyclin D1	1.3	1.1	2.5	-1.6	0.00062
INSIG1	insulin induced gene 1	1.3	1.5	1.3	2.8	0.0057
FGF10	fibroblast growth factor 10	1.2	1.2	-2.6	1.0	0.0031
DDIT3	DNA-damage-inducible transcript 3	1.2	-2.1	-1.8	-1.2	0.0036
C3	complement component 3	1.0	-1.4	2.1	-1.3	0.00052
PNPLA2	patatin-like phospholipase domain containing 2	-1.0	-1.6	-1.4	-1.2	0.0049
ABHD5	αβ hydrolase domain containing 5	-1.0	-1.4	-2.0	-1.1	0.00059
GPR109A	G protein-coupled receptor 109A	-1.2	-4.5	-10.6	-2.7	2.68E-08
RARα	retinoic acid receptor, alpha	-1.3	-2.4	1.1	-1.5	3.42E-05
RETNLα	resistin like alpha	-1.3	-1.1	3.0	-10.8	8.46E-08
ARL4A	ADP-ribosylation factor-like 4A	-1.3	1.3	-2.0	1.0	3.30E-05
EIF4EBP2	eukaryotic translation initiation factor 4E binding protein 2	-1.4	-1.6	-1.6	-1.2	0.0024
LAMA4	laminin, alpha 4	-1.4	-1.4	-1.4	-1.2	0.0065
C/EBPα	CCAAT/enhancer binding protein, alpha	-1.5	-1.4	-1.3	1.1	0.0062
FABP4	fatty acid binding protein 4, adipocyte	-1.5	-2.6	-1.2	-1.3	0.0026
AR	androgen receptor	-1.8	-2.0	-1.5	-1.8	0.00026
LEP	leptin	-2.0	-1.8	1.0	1.6	0.0017
IL6	interleukin 6	-2.0	-2.1	-20.5	-5.6	8.65E-07

The list includes the genes that change with age in both bone marrow and epididymal adipocytes. The set of the 27 regulated genes was selected on the basis of their fold changes across age in both adipocyte populations sorted using Partek Genome Suit software and IPA. The significance of differences was measured by two-way ANOVA. A low value in *p *indicates the gene is differentially expressed with respect to age. The comparison included 14-month (14M) and 18-month-old (18M) compared to 6-month-old.

## Discussion

Obesity accelerates the progression of aging and age-related metabolic disorders such as insulin resistance and type 2 diabetes [22,23]. The contribution of the heterogeneity of adipocytes to aging and age-related metabolic syndrome has been studied in subcutaneous and visceral adipose tissue [1,4]. Adipocytes and osteoblasts originate from MSCs within the bone marrow, where there is a reciprocal relationship in the development along each lineage [5-7]. During aging, increased bone marrow adipogenesis compromises hematopoietic stem cell differentiation and bone remodeling [24]. This study represents the first attempt to characterize primary bone marrow adipocytes by gene profiling and to compare their gene profiles with epididymal white adipocytes in response to aging. We observed a significant increase in fat infiltration in the bone marrow in 14-month-old male mice, which was accompanied by an increase in circulating insulin and a decrease in osteocalcin levels, reflecting some of the metabolic changes that occur with age.

### Differential gene profiling of bone marrow adipocytes

Microarray analysis of gene expression by gene ontologies and functional pathways allowed us not only to identify genes differentially expressed in bone marrow adipocytes, but also genes whose expressions were significantly altered during aging. Recently, Majka *et al *performed a microarray analysis of bone marrow progenitor-derived adipocytes which were purified from peripheral adipose depots of transplanted recipient mice by flow cytometry [20]. Their characterization showed that bone marrow progenitor-derived adipocytes are different from conventional white adipocytes with low expression levels of leptin, mitochondrial and peroxisomal capacity and high expression of inflammatory genes. The study design was different from the current study because their study examined bone marrow progenitor-derived adipocytes isolated from visceral or subcutaneous depots, whereas our study examined primary adipocytes isolated directly from bone marrow. Even with this major difference in design, our results with primary bone marrow adipocytes directly isolated from the bone marrow of aging mice agree remarkably well with their results. Global array analysis by PCA (Principal component analysis) mapping showed a clear separation between bone marrow and epididymal adipocytes. Previous studies have shown that macrophages express several genes classically associated with adipocytes, such as FABP4, Plin1 and PPARγ [25]; yet there are distinctions between adipocytes and macrophages. For instance, adipocytes do not express the macrophage specific cell surface marker CD11b (Mac-1α), while macrophages do not express adiponectin [26]. The primary adipocytes isolated from the bone marrow in our studies are characterized by low CD11b expression and high adiponectin (>3-fold) expression as confirmed by quantitative PCR. Expression levels of adipocyte-specific genes (Plin1, FABP4, leptin and Plin2) were also higher in the isolated bone marrow adipocytes than bone marrow stromal cells, thus confirming that the populations of cells studied are adipocytes. CD11b staining also confirmed that there were similar degrees of contamination of adipocytes by monocyte/macrophages in preparations from bone marrow and epididymal adipocytes. In our study, adipocyte specific genes (PPARγ, FABP4, Plin1, adipsin and leptin) were expressed at significantly lower levels in bone marrow adipocytes compared with epididymal white adipocytes, whereas the expression levels of inflammatory genes such as IL-6 and TNFα were found to be higher in bone marrow adipocytes compared with epididymal white adipocytes. The increased expression of proinflammatory cytokines in bone marrow adipocytes might be associated with a high level of macrophage infiltration within the aged bone marrow environment; however, as mentioned, we did not detect significant differences in macrophage contamination of the adipocyte preparations isolated from bone marrow versus epididymal depots. Interestingly, while leptin was expressed at a relatively lower level in bone marrow adipocytes than in epididymal adipocytes, the leptin receptor (LEPR) was more highly expressed in bone marrow adipocytes. In addition, expression of genes involved in fatty acid synthesis and fatty acid oxidation were lower in bone marrow compared with epididymal adipocytes, whereas genes associated with decreased mitochondrial function, such as TNFα, p53, BCL2L11 and IL-1β, were significantly higher in bone marrow compared with epididymal adipocytes. Furthermore, we observed that the expression of a group of genes associated with early adipocyte differentiation, including CEBP/β, KLF4 and EGR1&2, INHBA and S6K1, were higher in bone marrow adipocytes than epididymal adipocytes [27-29], indicating that bone marrow adipocytes are in an early stage of adipocyte differentiation. This observation also is supported by low expression of Plin1 and relatively higher expression of Plin2 in bone marrow adipocytes (14-month-old), as Plin2 is expressed early in adipocyte differentiation [30]. Previous studies also indicated that fat cell progenitors, preadipocytes, resemble the phenotype of macrophages. Preadipocytes account for 15 to 50% of cells in fat tissues [31]. Aging increases the numbers of preadipocytes and the production of cytokines PAI, IL-6 and other proinflammatory cytokines [1,32]. Thus, it is possible that the presence of newly differentiated adipocytes and fat cell progenitor cells might contribute to the differential expression patterns observed in bone marrow adipocytes.

### Age-related gene changes in bone marrow and epididymal adipocytes

Aging is characterized by fat redistribution with increased visceral fat and relative loss of subcutaneous fat in humans [22]. Microarray analysis of age-related gene expression in white adipose tissue (WAT) has been reported in rodents [33,34]. Linford et al reported that aging had a significant effect on gene expression in adipose tissues, particularly genes involved in PPARγ-dependent adipogenesis and lipid metabolism declined with age. Others have suggested that expression levels of key adipogenic transcription factors, C/EBPα, C/EBPδ and PPARγ, are lower in differentiating adipocytes isolated from old compared to young rats [35]. Here we observed a differential expression between bone marrow and epididymal white adipocytes with age. While PPARγ and C/EBPβ significantly increased in 14-month-old then decreased or did not change in 18-month-old bone marrow adipocytes, PPARγ increased and C/EBPβ decreased at 14 and 18 months in epididymal adipocytes. Global analysis of pathways associated with adipogenesis and lipolysis showed that expressions of genes within these pathways are generally increased in 14-month-old bone marrow adipocytes, whereas they are generally decreased in epididymal adipocytes. The observation that the expression pattern of some genes appears to be most highly affected at 14 months is possibly related to changes in body weight and fat mass, as body weights were highest at 14-months (Table 1) and declined at 18 months (36.5 ± 1.6 gm). Moreover, it is possible that age might have an even greater impact on differences in gene expression patterns if older animals (24 or 30 months) were examined.

A decrease in mitochondria function is associated with aging in heart, muscle, liver and white adipocytes in animals [36-39]. A decline in mitochondria function is highly associated with an increase in inflammation and apoptosis [40,41]. Linford et al reported that the most highly induced gene changes in adipose tissue and heart with aging in rats are genes associated with inflammation [33]. Our data are in agreement with previous reports demonstrating an increase in inflammatory genes in both adipocyte populations with age, and in particular in the bone marrow, since the pathway analysis indicated that there were more genes altered with age in bone marrow adipocytes. We further examined whether the genes known to be involved in aging were altered in adipocytes. For examples, SOD2 (superoxide dismutase 2) and Sirt1 (sirtuin 1), which are associated with mitochondrial aging [42], were significantly decreased in epididymal adipocytes with age, whereas these changes were not seen in bone marrow adipocytes. However, overall there was generally a similar expression pattern observed in both bone marrow and epididymal adipocytes in response to aging, but age has a greater impact on global gene expression in epididymal than in bone marrow adipocytes.

### Potential functional implications of gene expression profile of bone marrow adipocytes

In view of the physical proximity of adipocytes and osteoblasts within the bone marrow and the inverse relationship between bone mass and bone marrow adiposity, it seems reasonable to speculate that adipocytes can directly influence bone remodeling. Indeed, we have shown that adipocytes can modulate key metabolic functions of osteoblasts in co-culture through the release of secretory products (50). Daley et al suggest that adipocyte-secreted cytokines TNFα and adiponectin inhibit hematopoietic progenitor cell expansion within the bone marrow and also affect bone remodeling [26]. In this regard it is noteworthy that Wdnm1-like, an adipocyte-secreted protein, is expressed 3-4 fold higher in bone marrow adipocytes. Smas et al reported that Wdnm1-like is selectively expressed in adipose and liver and increases matrix metalloproteinase 2 (MMP2) activity [43]. It is possible that bone marrow adipocytes, through the release of Wdnm1-like, might contribute to extracellular remodeling within the bone marrow and mediate cross-talk between adipocytes, mesenchymal and hematopoietic stem cells. In addition to Wdnm1-like protein, we identified a number of genes that could potentially affect adipose-derived signaling to bone and whose expressions are increased in bone marrow adipocytes. For instance, the expression levels of oncostatin M (OM), SFRP4, ABCB4, TNFα, TGFβ1, GPR109A and IL-6 are markedly increased in bone marrow adipocytes. Whereas the actions of cytokines, such as OM, TNFα, and IL-6, and growth factors, such as TGFβ1, on bone are well described [44,45], SFRP4 is a phosphatonin that inhibits both tubular phosphate reabsorption and Wnt signaling, and whose expression has been reported to increase during adipose differentiation of MSCs and to be associated with lower bone density in mice [46-48]. Moreover, transgenic expression of SFRP4 in osteoblasts results in lower bone density [49]. ABCB4, also known as multidrug resistance P-glycoprotein 3, translocates phosphatidylcholine (PC) from the inner to the outer plasma membrane, thus functioning as a PC flippase; mutations in ABCB4 cause cholestatic liver disease [50]. Though no specific associations of ABCB4 with bone abnormalities are known, it is possible that adipocyte-derived PC might be supplied to and influence differentiation of MSCs or osteoblasts. GPR109A is the receptor for nicotinic acid, whose endogenous ligand appears to be a product of fatty acid oxidation, β-hydroxybutyrate [51]. This would reduce the local release of possible PPARγ or other receptor ligands or pro-ligands and potentially have a beneficial impact on bone. Therefore, the gene expression profile of bone marrow adipocytes is such that it could lead to both positive and negative effects on bone. The gene expression profile observed in 14-month-old bone marrow adipocytes was accompanied by elevated circulating serum insulin, glucose, adipocytokines (leptin, adiponectin) and reduced osteocalcin. The regulation of osteocalcin secretion by insulin has been shown in recent studies [16]. Therefore, it is possible that the increase in adipogenesis in the bone marrow of 14-month-old mice induces a negative influence on MSC lineages, thereby leading to a decline in osteoblast function, which could, in part, be attributed to hyperinsulinemia.

## Conclusions

Taken together, comparison of gene expression profiles in bone marrow adipocytes with epididymal adipocytes indicates that bone marrow adipocytes express adipocyte-specific genes, but appear to have a gene expression pattern that distinguishes them from epididymal adipocytes. Primary adipocytes within the bone marrow are characterized by low expression levels of adipocyte-specific genes and high levels of genes associated with early adipocyte lineage. However, adipocytes from both depots display common pathways and generally similar alterations in gene expression in response to aging. In depth research into the functional role of bone marrow adipocytes will be important for uncovering age related changes in bone, hematopoiesis and metabolic functions.

## Methods

### Experimental animals and isolation of adipocytes

Bone marrow adipocytes and epididymal white adipocytes (n = 6-10 animals per group) were isolated from male C57BL/6J mice (6-months, 14-months and 18-months of age). All mice were housed in temperature-controlled conditions on a 12-h light, 12-h dark cycle, and were fed standard chow. All procedures were in accordance with institution guidelines and approved by the institutional animal care and use committee of the VA Palo Alto Health Care System. Briefly, both femurs and tibias were collected after mice were sacrificed. Bones were cleaned and rinsed with 75% ethanol and DEPC water to eliminate surrounding fat and muscle cells. Fresh bone marrows were flushed out with PBS containing 1% fatty acid-free BSA and 1% RNAase and DNAase-free water using a 25-gauge needle from femurs and tibias. Red blood cells were lysed using red cell lysis buffer. After centrifugation at 3000 rpm for 5 min, floating adipocytes were collected from bone marrow stromal cells and then were washed with PBS buffer three times. In addition to the bone marrow, primary adipocytes were isolated from epididymal white adipose tissue (WAT) as described previously [52]. Briefly, epididymal WAT was removed from mice and minced with scissors into 2 ml Kreb-Ringer HEPES buffer supplemented with 3% BSA. Tissues were digested with collagenase type I (1mg/ml) for 40 min at 37°C in a 250 rpm shaker and adipocytes then isolated by flotation.

### Histology

Distal femurs isolated from 6-month-old and 14-month-old C57BL6/J male mice were decalcified in 4% EDTA and paraffin embedded following manufacturer's standard procedures (Histion, Everett, WA). Bones were sectioned in the sagittal plane to obtain cross sections of the distal femur and stained with hematoxylin and eosin (H&E). Fields were taken from distal femur sections of 6-month-old and 14-month-old mice and adipose areas calculated using ImagePro software.

## Microarray analysis

### RNA isolation, purification and array procedures

Total RNA was extracted using Trizol (Life Technologies, Grand Island, NY, USA) and chloroform followed by purification on an RNeasy MinElute column (QIAGEN, Valencia, CA, USA). Three pooled RNA preparations were generated from 6-10 animals due to the low yield of bone marrow adipocytes during isolation. RNA quality was verified using an Agilent Bioanalyzer (Agilent technologies, Palo Alto, CA, USA). Total RNA was biotin-labeled and hybridized to the GeneChip Mouse Gene 1.0 ST Array platform (Affymetrix, Santa Clara, CA, USA) with three RNA preparations per age group. The Protein and Nucleic Acid Microarray Facility at Stanford University carried out processing of DNA arrays according to standard protocols from the Affymetrix GeneChip^® ^Whole Transcript (WT) Sense Target Labeling Assay. This assay is designed to generate amplified and biotinylated sense-strand DNA targets from the entire expressed genome without bias. This assay and associated reagents have been optimized specifically for use with the GeneChip^® ^ST Arrays where "ST" stands for "Sense Target" and the probes on the arrays have been selected to be distributed throughout the entire length of each transcript. The microarray data files have been submitted to the Gene Expression Omnibus (GEO) and the accession number is GSE25905.

### Statistical analysis

The raw data from microarrays were analyzed using Partek^® ^Genome Suite software, version 6.3 Copyright^© ^2008 (Partek Inc., St. Louis, MO, USA). Briefly, Affymetrix .CEL files were processed to generate gcRMA (robust multi-array average) values. This step was followed by quantile normalization and log2 transformation to represent gene expression levels. Samples were grouped into cell type (bone marrow adipocytes vs. epididymal white adipocytes) and age (6-month (6M), 14-month (14M) and 18-month (18M)). ANOVA was performed including age and cell type interaction to generate the lists of differentially expressed genes comparing the bone marrow adipocytes with epididymal white adipocytes with age. There were three gene chips for each group. A total of 18 individual arrays contributed to the analyses. Probe sets with a fold-change 2.0 and adjusted *p*-value < 0.05 were considered differentially expressed between two cell types at each age group. The Benjamini-Hochberg false discovery rate (FDR) method was used for false positives. A corrected p-value cutoff of 0.05 was used to select the regulated genes with the lowest FDR. Partek^® ^Genome Suite was used as the first step for quality control (QC) of the data on all the samples with two methods, Pearson correlation and Principal Component Analysis (PCA). PCA was performed as a global view of sample clustering, which is related to the total variance in gene expression for all genes. Normalized expression values for all genes were analyzed. A selection of 3918 genes (13%) of the 28853 well-characterized mouse genes in Mouse Gene 1.0 ST array was differentially expressed between the two adipocyte populations. This selection was based on adjusted p < 0.05 and fold change ± 2.0. These 3918 genes were assigned to biologically meaningful gene ontology (GO) categories using GO enrichment analysis (Partek^® ^Genome Suite software, version 6.3).

Statistical analysis of metabolic parameters was performed using Graphpad Prism 4.0. Age dependent changes were statistically analyzed by one-way ANOVA (repeated measures for within subject samples). Data are presented as mean ± SE.

### Pathway Analysis

For each comparison a list of differentially expressed genes was generated. The gene lists, along with associated expression or fold-change values, were further analyzed using Ingenuity Pathway Analysis (Ingenuity system, Inc, Redwood City, CA, USA) to identify differentially expressed pathways between bone marrow adipocytes and epididymal white adipocytes with age. The list of significantly regulated genes selected by the microarray analysis described above was loaded in IPA with the following criteria: reference set: Mouse 1.0 ST Gene assay; direct and indirect relationships included filtered by species (mouse) and by tissue (adipose). Then IPA computed the data to generate significant networks of genes that are associated with particular biological functions, diseases and signaling pathways.

### Analysis of gene expression by real-time RT-PCR

Total RNA was extracted from cells with TriZol reagent (Invitrogen, Carlsbad, CA, USA) according to the manufacturer's protocol. Total RNA was reverse transcribed with random hexamers by using reverse transcriptase (Invitrogen, Carlsbad, CA, USA). Expression of selected adipose genes in preparations of bone marrow cells, adipocytes isolated from bone marrow and adipocytes isolated from epididymal white adipose tissue. A set of original RNA from the same animal was re-amplified to aRNA, and then converted to cDNA. After cDNA synthesis, mRNA expression levels were determined by using SYBR green qPCR supermix (ABI, Foster City, CA, USA). Expression levels of mRNA were analyzed by quantitative real-time PCR (Prism 7900 Sequence Detection System, Applied Biosystems (ABI), Foster City, CA, USA). Relative gene expression was calculated using the comparative threshold method (2 − ΔΔCt) [53]. Relative fold change was normalized to endogenous 18S and bone marrow stromal cells. Data are presented as Log2 of fold change. Primers are listed in Additional File 2, **Table S1**.

### Determination of insulin and adipokine concentrations by Luminex ELISA

Serum obtained from mice was analyzed for adipokines and bone panel measurements using a multiplex mouse adipokines assay (Mouse adipocyte panel, Millipore, Bedford, MA, USA), and detected by Luminex xMAP method (Luminex 200, Millipore, Bedford, MA, USA). Insulin and adipokines including adiponectin, leptin, resistin, and bone markers osteocalcin, RANKL and osteoprotegerin were measured in 6-month and 14-month-old mice in the fed state.

### Immunostaining

Percentage of monocytes was determined by counting the number of CD11b positive cells per 100 cells. Approximately 200 to 400 cells were counted in each experiment. Freshly isolated adipocytes from bone marrow or epididymal adipose tissue were fixed with 3.7% paraformaldehyde in PBS for 1 h at room temperature. Fixed cells were incubated with either BODIPY 493/503 (1:500, 1 mg/mL, Molecular Probe, Carlsbad, CA, USA) or PE (phycoerythrin) conjugated anti-mouse CD11b (Intergrin a, Mac-1α, eBioscience Inc, San Diego, CA, USA) at 0.124 μg per million cells in 100 μL total staining volume and polyclonal anti rabbit perilipin antibodies (1:200; a kind gift of Dr. Andrew Greenberg, Tufts University) in 1% blocking solution for 1 h. Following 1 h incubation of Alexa 555 (red) conjugated secondary antibodies at a dilution of 1:800 at room temperature, the stained cells were washed three to four times with PBS and observed using a Zeiss Axio Observer A1 microscope (Carl Zeiss, Thornwood, NY, USA).

## Authors' contributions

LFL designed and performed experiments and wrote the manuscript; WJS, MU and SP performed experiments; FBK designed the experiments and wrote the manuscript.

## Supplementary Material

Additional file 1 **Figure S1: Evaluation of gene expression microarray data sets**. RNA from bone marrow and epididymal white adipocytes (three samples each age group) was converted to cDNA, labeled and hybridized to Affymetrix Mouse Gene 1.0 ST arrays. Scanned data were imported into Partek Genomics Suit Software and normalized using RMA algorithim. A plot of frequency versus signal value is shown for each dataset. Click here for file

Additional file 2 **Table S1: List of primers for qRT-PCR**. Click here for file
