# Supplementary figures and images for: Characterization of age-related gene expression profiling in bone marrow and epididymal adipocytes
Source: BMC Genomics. 2011 May 5;12:212. doi: 10.1186/1471-2164-12-212 (PMC3113784; doi:10.1186/1471-2164-12-212)

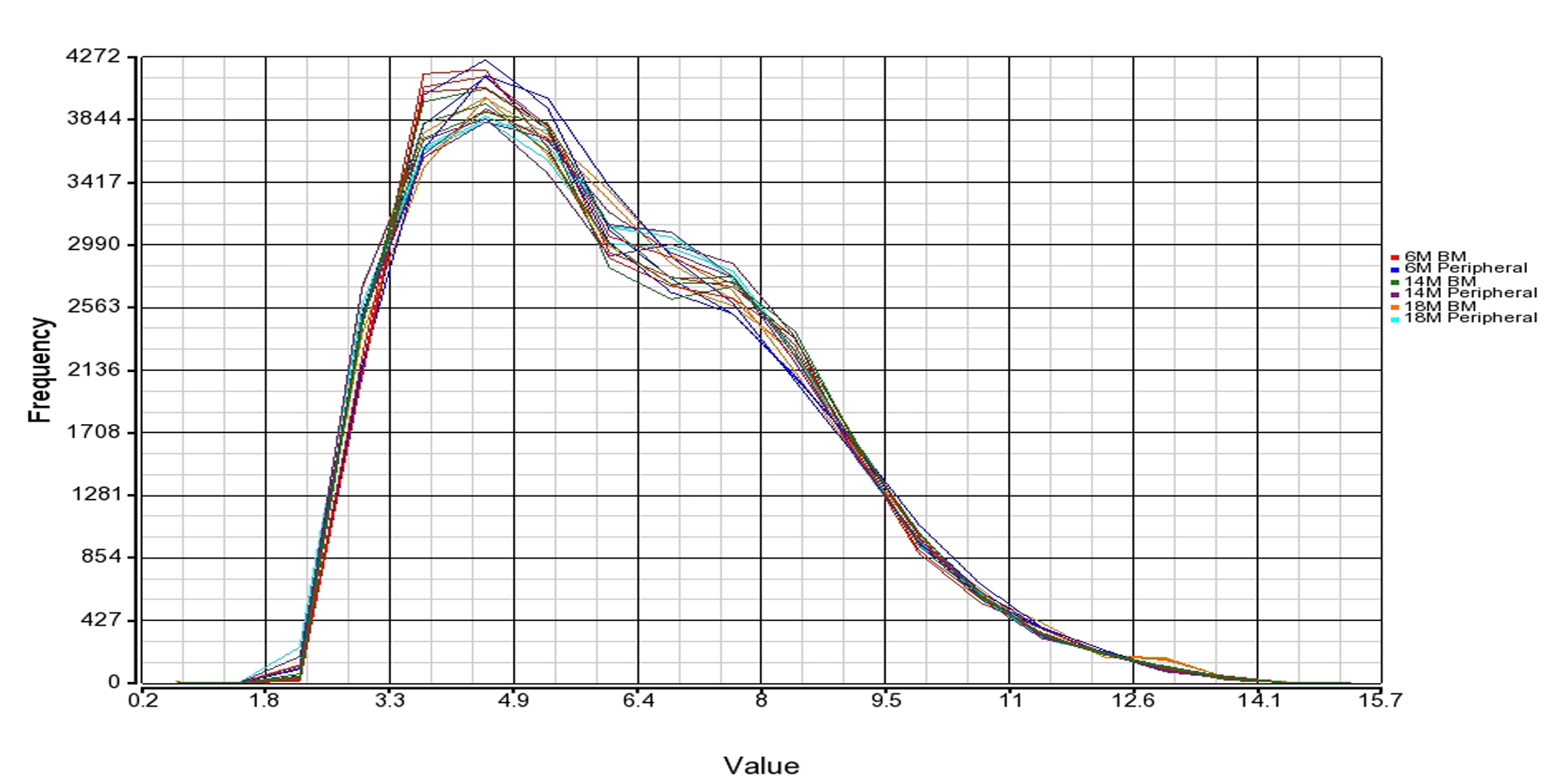

Supplement: Additional file 1 — Figure S1: Evaluation of gene expression microarray data sets. RNA from bone marrow and epididymal white adipocytes (three samples each age group) was converted to cDNA, labeled and hybridized to Affymetrix Mouse Gene 1.0 ST arrays. Scanned data were imported into Partek Genomics Suit Software and normalized using RMA algorithim. A plot of frequency versus signal value is shown for each dataset. [file 1471-2164-12-212-S1.TIFF]
