# Supplementary material for: Characterization of age-related gene expression profiling in bone marrow and epididymal adipocytes
Source: BMC Genomics. 2011 May 5;12:212. doi: 10.1186/1471-2164-12-212 (PMC3113784; doi:10.1186/1471-2164-12-212)
Supplement: Additional file 2 — Table S1: List of primers for qRT-PCR. [file 1471-2164-12-212-S2.DOC]

**Table S1.** List of primers for RT-PCR

| Gene symbol | Entrez gene name | Gene Assession number |  | **Sequence** (5' -> 3') | **Length** | **Tm** |
| --- | --- | --- | --- | --- | --- | --- |
| FABP4, AP2, ALBP | Fatty acid binding protein 4, adipocyte | NM_024406 | Forward Primer | GCGTGGAATTCGATGAAATCA | 21 | 62.4 |
|  |  |  | Reverse Primer | CCCGCCATCTAGGGTTATGA | 20 | 62.5 |
| Lep, OB | Leptin | NM_008493 | Forward Primer | GGGCTTCACCCCATTCTGA | 20 | 61.6 |
|  |  |  | Reverse Primer | GCCCACCAACTTCGGAATC | 21 | 61.2 |
| PPARγ | Peroxisome proliferator-activated receptor gamma | BC021798 | Forward Primer | TGCGAGTGGTCTTCCATCAC | 20 | 63 |
|  |  |  | Reverse Primer | GTCGTTGAACTCCTCGGTCT | 20 | 61.2 |
| Plin 1 | Perilipin 1 | XM_133574 | Forward Primer | CATCTCTACCCGCCTTCGAA | 20 | 62.7 |
|  |  |  | Reverse Primer | TGCTTGCAATGGGCACACT | 19 | 60.1 |
| Plin 2, Adipopilin | Perilipin 2 | NM_007408 | Forward Primer | AGCCAACGTCCGAGATTGTT | 20 | 60.2 |
|  |  |  | Reverse Primer | CACATCCTTCGCCCCAGTT | 19 | 60.2 |
| ADIPOQ, ACRP30 | Adiponectin | NM_009605 | Forward Primer | CGGCAGCACTGGCAAGTT | 18 | 60.9 |
|  |  |  | Reverse Primer | CCGTGATGTGGTAAGAGAAGTAGTAGA | 26 | 60.2 |
| CEBP/β | Ccaat/enhancer binding protein beta | X62600 | Forward Primer | AAGCTGAGCGACGAGTACAAGA | 22 | 60.4 |
|  |  |  | Reverse Primer | GTCAGCTCCAGCACCTTGTG | 19 | 60.2 |
